# Does the pressure dependence of kinetic isotope effects report usefully on dynamics in enzyme H‐transfer reactions?

**DOI:** 10.1111/febs.13193

**Published:** 2015-01-29

**Authors:** Robin Hoeven, Derren J. Heyes, Sam Hay, Nigel S. Scrutton

**Affiliations:** ^1^ Manchester Institute of Biotechnology and Faculty of Life SciencesThe University of ManchesterUK

**Keywords:** dynamics, flavoprotein, hydrogen transfer, pressure, quantum tunnelling

## Abstract

The temperature dependence of kinetic isotope effects (KIEs) has emerged as the main experimental probe of enzymatic H‐transfer by quantum tunnelling. Implicit in the interpretation is a presumed role for dynamic coupling of H‐transfer chemistry to the protein environment, the so‐called ‘promoting motions/vibrations hypothesis’. This idea remains contentious, and others have questioned the importance and/or existence of promoting motions/vibrations. New experimental methods of addressing this problem are emerging, including use of mass‐modulated enzymes and time‐resolved spectroscopy. The pressure dependence of KIEs has been considered as a potential probe of quantum tunnelling reactions, because semi‐classical KIEs, which are defined by differences in zero‐point vibrational energy, are relatively insensitive to kbar changes in pressure. Reported combined pressure and temperature (*p*‐*T*) dependence studies of H‐transfer reactions are, however, limited. Here, we extend and review the available *p*‐*T* studies that have utilized well‐defined experimental systems in which quantum mechanical tunnelling is established. These include flavoproteins, quinoproteins, light‐activated enzymes and chemical model systems. We show that there is no clear general trend between the *p*‐*T* dependencies of the KIEs in these systems. Given the complex nature of *p*‐*T* studies, we conclude that computational simulations using determined (e.g. X‐ray) structures are also needed alongside experimental measurements of reaction rates/KIEs to guide the interpretation of *p*‐*T* effects. In providing new insight into H‐transfer/environmental coupling, combined approaches that unite both atomistic understanding with experimental rate measurements will require careful evaluation on a case‐by‐case basis. Although individually informative, we conclude that *p*‐*T* studies do not provide the more generalized insight that has come from studies of the temperature dependence of KIEs.

**Abbreviations**
AADH: aromatic amine dehydrogenase
KIE: kinetic isotope effect
MR: morphinone reductase
PCET: proton coupled electron transfer
Pchlide: protochlorophyllide
PETNR: pentaerythritol tetranitrate reductase
POR: protochlorophyllide oxidoreductase
*p*‐*T*: pressure‐temperature
RHR: reductive half reaction
TEA: tetramethylammonium chloride

## Isotope effects and dynamics in enzyme catalysed reactions

A central paradigm in biochemistry is that protein function is defined by structure. However, in solution proteins are inherently dynamic molecules, exhibiting motions on timescales ranging from bond stretches (~ 10^3^ cm^−1^; fs) through to slow domain motions and normal mode vibrations (< 1 cm^−1^; ms). An important open question in enzymology remains the role of such dynamics, and whether motions/vibrations on timescales faster than turnover (i.e. *k*_cat_) can couple to chemical steps during catalysis (i.e. to the reaction coordinate) 1. There has been good progress using NMR approaches in establishing the role of ms–ns dynamics such as loop opening/closing during enzyme turnover 2, 3, but direct evidence for the coupling of faster (sub‐ns) dynamics to chemistry remains illusive and controversial 1, 4, 5, 6, 7, 8 and is inferred largely on the anomalous temperature dependencies of primary kinetic isotope effects (KIEs; e.g. *k*_H_/*k*_D_) 9, 10, 11, 12. The role of fast dynamics remains an important question, because motions on similar timescales to chemistry (ps–fs; specifically, the time required to traverse the transition state) have the potential to profoundly affect the reaction outcome, and thus offer a means to control (enzyme) reactivity 1, 13, 14.

The potential importance of fast motions in H‐transfer reactions where quantum mechanical tunnelling is a feature of the reaction has been debated intensely 1, 5, 6, 7, 15. KIEs and analysis of their temperature dependence are now established as a general approach to investigate quantum mechanical tunnelling reactions in enzymes 6, 9, 10, 16. In the absence of complicating issues (e.g. reaction branching 17), inflated KIEs (values above the semi‐classical limit of ~ 7 at 298 K) are generally taken to be a definitive hallmark of quantum mechanical hydrogen tunnelling 18. Quantum tunnelling is also a feature of many reactions in which intrinsic KIEs are numerically below the maximum value (attributed to the difference in zero point vibrational energies of the C–H and C–D bonds) predicted by semi‐classical transition state theory 19. These findings have been rationalized in the context of simple Marcus‐like (vibronic) models of H‐transfer 20, 21, 22, 23, which have been used widely by the experimental enzymology community to study quantum mechanical tunnelling and the inferred importance of dynamics in H‐transfer reactions. The limitations of these models have been discussed, and alternative explanations of the temperature dependence of KIEs have been advanced 4, 5, 24, 25, 26, but semi‐quantitatively these simple vibronic models have enabled comparisons to be made of tunnelling and inferred dynamics in enzymes, for example, across a reaction series where active site structure and dynamics are altered by site‐directed mutagenesis 27, 28 or where studies are made with a single enzyme using multiple substrates 29, 30. Despite their limitations, vibronic models have provided some useful insight into tunnelling and the inferred importance of dynamics 20, 21, 22, 23, especially for estimating the frequencies of inferred compressive dynamics/promoting motions and donor–acceptor distances 11, 26, 27, 28, 31. Experimental studies of this type are best interpreted alongside more detailed atomistic simulations of the reaction chemistry where possible, to provide quantitative insight into the reaction free energy barrier, the extent of tunnelling, and coupling of the protein environment to the reaction coordinated 7, 19, 32, 33, 34, 35.

More recently, analysis of the temperature dependence of primary KIEs in relation to quantum tunnelling and the inferred importance of dynamics have taken on a new direction by employing mass modulated (‘heavy’) enzymes. For many years, investigators have exploited the use of stable isotope‐labelled proteins (typically labelled with ^2^H, ^13^C and/or ^15^N) as an experimental tool, particularly in the NMR and vibrational spectroscopy communities (e.g. ^13^C and ^15^N are used as NMR probes, whereas amino acid isotopic labelling is used in FTIR experiments to shift vibrational spectra and to aid in peak assignment). The implicit assumption has generally been that isotopic labelling does not significantly perturb protein function. However, D_2_O has been shown to promote rigidification and unfolding of some proteins 36, whereas perdeuteration has long been known to significantly reduce the rate of turnover of alkaline phosphatase 37. Recently, Schramm and colleagues showed that isotopically labelled ‘heavy’ purine nucleoside phosphorylase and HIV‐1 protease enzymes have measurably slower reaction kinetics 38, 39. These data were interpreted in terms of the Born–Oppenheimer approximation, in which increased protein mass (due to labelling) alters bond vibrational frequencies without affecting electrostatic properties of the enzyme (ionizable protons were not labelled). The authors suggested that the lower frequency of (fs) bond vibrations in the ‘heavy enzymes’ may lead to a reduction in conformational sampling and thus chemical barrier crossing; the rate of reaction is proportional to the rate of barrier crossing. Clearly, the ‘heavy enzyme’ methodology can be used as a powerful tool to study enzyme dynamics and others have adopted this approach. We have extended this approach to perturb the temperature dependence of a KIE on the Old Yellow flavoenzyme pentaerythritol tetranitrate reductase (PETNR) catalysed hydride transfer reaction 40, although others have measured isotope effects on ‘heavy’ alanine racemase 41 and dihydrofolate reductase enzymes 33, 34. A common finding is that in each case, the catalysed reaction is slower in the ‘heavy’ enzyme suggesting that vibrational coupling of the protein to the reaction coordinate may be a general feature. However, important questions remain. Mass perturbation will affect all vibrations within the protein, so experimental observation of the timescale(s) of any vibrational coupling between protein and chemical coordinate is highly desirable in order to firmly establish the origin of the ‘heavy enzyme’ effect. Further, a computational study of a ‘heavy’ dihydrofolate reductase variant suggests that an increased dynamic coupling to the chemical coordinate is detrimental to dihydrofolate reductase catalysis 33. It is now timely to also consider whether the dynamic coupling of enzyme motions to the chemical coordinate is generally optimized (e.g. by evolution). ‘Heavy’ enzymes offer a more refined alternative to traditional mutagenesis approaches to study such questions and provide new experimental tools with which to explore the potential importance of dynamics/environmental coupling to the reaction coordinate.

## Pressure–temperature dependence of KIEs

An alternative and complementary experimental approach is to analyse the combined pressure–temperature (*p*‐*T*) dependence of KIEs with a view to correlating outcomes with *T* dependence studies of KIEs with conventional and mass‐modulated enzymes. However, the usefulness of *p*‐*T* dependence studies as a probe of environmental coupling/dynamics in enzymatic H‐tunnelling reactions is uncertain because of the limited subset of reactions that have been studied. Here, we review and extend *p*‐*T* dependence studies of primary KIEs for H‐transfer reactions catalysed by enzyme and simple model systems to investigate their general utility as experimental probes of dynamics. We provide first an overview of the theory that we have developed for analysis of KIEs as a function of pressure and temperature. We then discuss our recent *p*‐*T* dependence studies with selected flavoprotein and quinoprotein systems, which highlight the utility (and potential problems) of this approach. Finally, we present new *p*‐*T* dependence studies with the light‐activated enzyme protochlorophyllide oxidoreductase (POR) and explore relationships/correlations across multiple datasets (including those from non‐enzymatic model chemical studies) to understand how *p*‐*T* dependence studies can inform on tunnelling and/or environmental coupling, and to highlight the strengths and limitations of such an approach.

Although a number of vibronic models have been developed to describe H‐transfer by vibrationally assisted quantum tunnelling (20, 21, 22, 23 and below), the analysis of the temperature and/or pressure dependence of observed rate constants using transition state theory in parallel facilitates comparison with other work; the apparent activation enthalpy, Δ*H* ^**‡**^, and entropy, Δ*S* ^**‡**^ (or equivalent; i.e. *E*_a_ and *A*, respectively) are obtained from the Eyring or Arrhenius equations (Eqn (1)) 42, whereas the apparent activation volume, Δ*V* ^**‡**^, and activation isothermal compressibility, Δβ^**‡**^, are obtained from the pressure dependency (Eqn (2)) 23, 43, 44, 45.

(1) $$k_{\mathrm{obs}}\left(T\right)=k_{B}T/h\exp\left(\Delta S^{‡}/R\right)\exp\left(-\Delta H^{‡}/RT\right)=A\exp\left(-E_{a}/RT\right)$$

(2) $$k_{\mathrm{obs}}\left(p,T\right)=k_{0}\exp\left(-\Delta V^{‡}p/RT\right)\exp\left(\Delta \beta^{‡}p^{2}/2RT\right)$$

The temperature and pressure dependencies of KIEs can likewise be fitted to Eqns (1) and (2) by substituting KIE values for *k*_obs_. In this case, the temperature dependence of the KIE is described by the difference in the entropy of activation, ΔΔ*S* ^‡^ = Δ*S* ^‡D^ − Δ*S* ^‡H^ ~ *R*ln(−*A* ^H^/*A* ^D^) and the difference in the enthalpy of activation, ΔΔ*H* ^‡^ = Δ*H* ^‡D^ − Δ*H* ^‡H^ ~ Δ*E*_a_. Likewise, the pressure dependence of the KIE is described by KIE_0_ (the KIE extrapolated to zero pressure), the difference in the activation volume ΔΔ*V* ^‡^ = Δ*V* ^‡H^ − Δ*V* ^‡D^ and the difference in the activation isothermal compressibility ΔΔβ^‡^ = Δβ^‡H^ − Δβ^‡D^. A complication arises in that KIE_0_, (Δ)Δ*V* ^‡^ and (Δ)Δβ^‡^ may be significantly temperature dependent, so ideally rate constants and KIEs should be measured over a matrix of pressure and temperature values, i.e. a *p*‐*T* matrix 11, 46.

Isaacs *et al*. showed that H‐transfer reactions with a significant degree of quantum tunnelling of the transferred H could exhibit pressure‐dependent KIEs 44. Later, Northrop developed a model 43 for the pressure dependence of H‐transfer reactions with a small tunnelling component (*Q*), which is based on the Bell correction 18:

(3) $$\begin{array}{c} \text{KIE}{}_{\mathrm{obs}}\left(p,T\right)=\text{KIE}{}_{0}+{\text{KIE}}_{0}\left(Q^{H}/Q^{D}-1\right)\\ \text{exp}\left(-\Delta V^{Q}p/\text{RT}\right) \end{array}$$

Functionally, Eqn (3) is similar to Eqn (2) if (Δ)Δβ^‡^ is fixed to zero, because the observed KIE has an exponential dependence on pressure. Consequently Δ*V* ^Q^ and ΔΔ*V* ^‡^ should be comparable. More recently, we developed a model to account for the *p–T* dependence of H‐tunnelling reactions (Eqn 21 in Ref. 23) based on an approximate vibronic formulism, which can be expressed by:

(4) $$\begin{array}{c} {\text{KIE}}_{\mathrm{obs}}\left(p,T\right)\approx \;\text{KIE}{}_{0}\;\text{exp}\;\left(-2Ek_{B}T\;\left(\kappa_{0}+\Delta \kappa \cdot p\right)\right)\\ \text{exp}\left(E{\left(r_{0}-\Delta r\cdot p\right)}^{2}\right)\\ E=\;\left(\mu_{h}\omega_{h}-\mu_{l}\omega_{l}\right)/2\hbar \end{array}$$

In this case, the observed KIE has a similar pressure response as Eqn (2); i.e. the pressure dependence of ln(KIE_obs_) is a quadratic function of pressure.

A more general effect of pressure is to perturb pre‐existing equilibrium, favouring species with smaller volumes 43, 45. Consequently, if multiple conformational states (heterogeneity) are involved in the reaction (as has been proposed by some workers in the field 5, 25, 26, 47), then pressure may perturb rate constants and/or KIEs by perturbing the relative concentration(s) of reactive states 46, 48. In this case, the pressure dependence of the KIE may not be well defined, especially for KIEs measured under steady‐state turnover conditions in which measured KIEs are an average of those for individual conformational states, weighted to reflect the distribution of these states at a defined pressure 48.

## *p*‐*T* dependence of KIEs in flavoproteins

**Figure 1:**
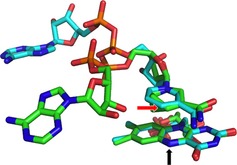
Overlay of the FMN and nicotinamide coenzyme within the active sites of NADH_4_‐bound MR (2R14.pdb, green carbons) and PETNR (3KFT.pdb, teal carbons) 30, 47. The nicotinamide C4 (hydride donor atom) is indicated with a red arrow and the FMN N5 (acceptor) with a black arrow. Hydrostatic pressure appears to reduce the C4–N5 distance due to compression roughly along the vertical axis 52, 53.

We initially examined the *p–T* dependence of the primary KIE on hydride transfer during the reductive half reaction (RHR) of the flavin‐containing enzyme morphinone reductase (MR) with the coenzyme NADH 11, 23. This reaction involves hydride transfer from the C4 R‐hydrogen of NADH to the N5 atom of FMN (Fig. 1) and can be observed directly using a (variable pressure) stopped‐flow instrument. The reaction transients reporting on the chemical step, display a primary and alpha secondary isotope effect and are consistent with transfer by quantum mechanical tunnelling 19, 49. The temperature dependence of the primary KIE has been described within the context of environmentally coupled (vibronic) Marcus‐like models for H‐transfer, and the potential importance of fast promoting motions to move the nicotinamide C4 H close to the flavin N5 to optimize H‐transfer has been inferred from these data 11, 28. More recently, we used mass modulated forms of the flavoenzyme pentaerythritol tetranitrate reductase (PETNR, which is structurally and functionally related to MR) to show that stable isotope labelling of the enzyme perturbs the temperature dependence of the primary KIE. This has been interpreted as establishing a causal relationship between fast motions and enzyme chemistry for hydride transfer from NADH to FMN in this class of enzyme 40.

In *p*‐*T* studies with MR, both the observed rate constants and primary KIE for hydride transfer from NADH to FMN were found to increase with pressure, whereas ΔΔ*H* ^‡^ was not significantly temperature dependent and the secondary KIE decreased with pressure 11, 50. Similar pressure dependencies of the rate and KIE on the RHR of PETNR with NADH and NADPH were observed 30. A positive correlation between ΔΔ*H* ^‡^ and ΔΔβ^‡^ across these three experiments led us to propose that ΔΔβ^‡^ may be an alternative probe of fast dynamics in enzymes 30, 51.

Numerical modelling of the *p*‐*T* dependence of the MR primary KIE using an environmentally coupled H‐tunnelling model has suggested, to a first approximation, that the experimental data are consistent with the donor–acceptor distance oscillating around an equilibrium separation (*r*_o_) 11, 23. It is implied in this analysis that the magnitude of the KIE can increase if the frequency of the oscillation (i.e. the inferred promoting motion) is allowed to increase while the equilibrium separation is simultaneously decreased. This modelling was used to rationalize the observed trend in KIE (and rate constant) for hydride/deuteride transfer as the hydrostatic pressure is increased. As pressure is increased, the inferred effect is to shorten the equilibrium separation between donor and acceptor. This increases the rate of hydride transfer – the transfer of which still involves quantum mechanical tunnelling – and leads to both a stiffening of (i.e. increase in oscillator force constant) and a reduction in distance sampling by the inferred promoting motion.

Numerical modelling implies that the effect of pressure is to reduce donor–acceptor distance in MR and PETNR. In both enzymes, the nicotinamide moiety of the NAD(P)H coenzyme stacks over the FMN isoalloxazine via a π–π stacking interaction (Fig. 1) giving rise to a transient long‐wavelength charge‐transfer species in stopped‐flow studies of the reductive half reaction. This charge‐transfer species can be stabilized using the coenzyme mimic NAD(P)H_4_ – a form of the coenzyme that is unable to transfer hydride from the coenzyme to FMN, but retains its ability to charge‐transfer with the FMN isoalloxazine ring. Pressure spectroscopy of the ternary complex of MR saturated with NADH_4_ has demonstrated a compression of the charge‐transfer bond at high pressure 52, which has been corroborated by molecular dynamics simulations of the MR–NADH complex 52, 53. We have also demonstrated that high pressure leads to a decrease in the observed α‐2° KIE on the pre‐steady‐state hydride transfer from NADH to FMN in MR 50. This was also rationalized as a reduction in macroscopic reaction barrier width for this reaction, and vibrational analysis by density functional theory of a simple active site model indicated that the decrease in the α‐2° KIE with pressure is attributed to a decrease in vibrational coupling between the NADH primary (transferred) and secondary hydrogens in the ‘tunnelling ready conformation’ 50.

Numerical models developed for MR (and PETNR) based on the extensive *p*‐*T* analysis of KIEs for MR provide a framework for understanding how modulation of donor–acceptor distances influences the hydride transfer process. Use of these models has been extended to studies with variant forms of MR in which donor–acceptor distances are changed by site‐directed mutagenesis in the coenzyme‐binding pocket 28. Likewise, the effects of using alternative coenzymes (e.g. NADH in place of NADPH, the natural coenzyme for PETNR) to modulate donor–acceptor distances and the force constant for inferred promoting motions have been discussed 28. What is clear is that sub‐Angstrom changes in donor–acceptor distance can have a major effect on the rate of hydride transfer, the KIEs obtained and the presumed importance (or otherwise) of promoting motions in facilitating the reaction. Detecting such small perturbations in donor–acceptor distance is experimentally challenging. Recent work, however, shows that ultrafast transient absorption spectroscopy of photoinduced electron transfer rates in NAD(P)H_4_‐bound MR and PETNR is a sensitive probe of donor–acceptor distance, providing a ‘kinetic ruler’ for probing small perturbations in donor–acceptor distance 54.

## *p*‐*T* dependence of KIEs for aromatic amine dehydrogenase

Our work with flavoprotein systems provided motivation to study the *p*‐T dependence of primary KIEs with the quinoprotein aromatic amine dehydrogenase (AADH). Our studies with MR and PETNR had suggested that pressure might be a useful general probe for both quantum tunnelling and compression of the reaction coordinate, and we were keen to investigate the generality of this finding with other systems such as AADH, which is known to catalyse proton transfer by quantum mechanical tunnelling 46. With the substrate tryptamine the KIE for proton/deuterium transfer in the reductive half reaction of AADH is large (~ 55) and variational transition state theory calculations/spectral density analysis from molecular dynamics simulations are consistent with this being a quantum mechanical tunnelling reaction, assisted by an inferred promoting vibration 31, 32. We have also explored the reaction with alternative substrates (*para*‐substituted phenylethylamines), mainly because the reaction kinetics are more readily accessed by the stopped‐flow method over a temperature range. In these cases, KIEs are smaller (~ 20–30, depending on reaction conditions) and show varying degrees of temperature dependence consistent with the coupling of promoting motions to the reaction coordinate 29. The *p*‐*T* dependence was recently studied with the substrate phenylethylamine, and this highlighted a complex response attributed to a pressure‐mediated anisotropic (de)compression of the enzyme 46. With AADH, increasing pressure was found to decrease the rate of proton transfer, but this is not attributed to significant changes in donor–acceptor distance across the pressure range. Constant‐pressure molecular dynamics simulations have indicated that the average radius of gyration <*R*_gyr_> for the AADH–phenylethylamine complex is, as expected, reduced at higher pressures. However, the effect of pressure on the structure of AADH is anisotropic – principal component analysis on the absolute change in atomic coordinates as a function of pressure revealed that the change in the average <*R*_gyr_> can be deconvoluted along three vectors, with the majority of the change in <*R*_gyr_> occurring in one dimension. Importantly, this vector is not aligned with the reaction coordinate and thus significant alteration (compression or decompression) of the reaction coordinate is not observed in AADH on changing pressure.

Our work with AADH has indicated that more complete understanding of pressure effects on KIEs in enzymes is also dependent on gaining atomistic understanding derived from molecular dynamics calculations of known structures, and ultimately through, for example, the use of a computation to generate an ensemble of reactive geometries followed by identification of a rigorous reaction coordinate. Importantly, our work established that a pressure‐dependent KIE is not necessarily a definitive hallmark of quantum tunnelling. With AADH we observed pressure‐independent KIEs even though proton transfer is known to occur, from the temperature dependence of the KIE, by quantum mechanical tunnelling. Thus, although semiclassical KIEs are expected to be pressure independent, KIEs for tunnelling reactions are variably pressure dependent and in general terms a pressure‐independent KIE cannot be used to rule out a tunnelling contribution.

## Model chemistry (ascorbate and ferricyanide)

**Figure 2:**
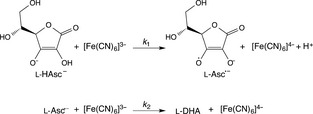
Ascorbate (l‐Asc^−^) oxidation by ferricyanide ([Fe(CN)_6_]^3−^) in aqueous solution occurs in two sequential one‐electron transfer reactions to produce dehydroascorbic acid (l‐DHA).

Recently, we analysed the *p*‐*T* dependence of the KIE on proton‐coupled electron transfer (PCET) during ascorbate oxidation by ferricyanide, and demonstrated that this reaction was consistent with vibrationally assisted tunnelling of the transferred proton 55. This model chemical system (Fig. 2) is a potential reference reaction for biochemical transformations catalysed by ascorbate peroxidases and cytochrome *b*_561_ proteins. Solvent isotope effects have been reported on the first kinetic step (oxidation of ascorbic acid by ferricyanide; *k*_1_) consistent with a PCET reaction. Temperature dependence studies have indicated that: (a) the reaction occurs by quantum mechanical tunnelling 18; (b) the KIE is temperature dependent, consistent with the promoting motions hypothesis 56, 57; and (c) the temperature dependence is strongly influenced by the solvent composition 57.

In extending this work, we determined the *p*‐*T* dependencies of ferricyanide reduction by ascorbate to experimentally evaluate the rate constant for PCET using rapid mixing stopped‐flow spectroscopy in H_2_O‐ and D_2_O‐buffered solutions 55. The temperature dependence of the KIE for PCET was found to depend strongly on the presence or absence of tetraethylammonum chloride (TEA) in the reaction buffer, being more pronounced in the presence of TEA. In the absence of TEA, the KIE was marginally temperature dependent; the KIE was, however, found to be significantly pressure dependent, consistent with transfer by quantum tunnelling. Of interest was the finding that, in the presence of TEA, the magnitude of the KIE was increased (beyond the maximum expected for semiclassical descriptions of H‐transfer), but its pressure dependence was negligible (although maintaining a strong temperature dependence). Like the *p*‐*T* dependence of the KIE on the AADH reaction (above), the data led us to conclude that, despite previous reports 43, 44, the absence of a pressure dependence of a KIE on H‐transfer is not evidence for a lack of tunnelling during the reaction. Instead, we interpreted the combined *p*‐*T* dependence of the ascorbate KIE such that that: (a) the PCET involves quantum mechanical tunnelling of the transferred proton, because the KIE > 7 in the presence of TEA; and (b) the presence of TEA influences vibrational coupling of H‐transfer to the environment (i.e. the apparent promoting motion), reflected in changes to the temperature and pressure dependencies of the KIEs.

Because the kinetic properties of the model chemistry are readily modulated by changes in solvent composition, this reaction is suited to exploring in detail the potential role of promoting motions in quantum tunnelling in ways not readily achievable with enzyme systems. In many enzyme systems, changes to the temperature and pressure dependence will often require modifications in the immediate protein environment using site‐directed mutagenesis, which does not allow one to alter the tunnelling characteristics in a relatively straightforward and predictable way. Studies with model systems might, therefore, add significantly to the body of data in the literature and provide tractable systems with which to rigorously test the promoting motions hypotheses often discussed in the enzyme tunnelling literature.

## Light‐activated POR

In contrast to the thermally activated systems described above, the light‐driven chlorophyll biosynthetic enzyme POR provides a unique opportunity to trigger catalysis by using a single pulse of light 58, 59. POR catalyses the reduction of the C17–C18 double bond of the protochlorophyllide (Pchlide) substrate and has become an important model system for studying the mechanisms of H‐transfer reactions 58, 59. Following illumination, a hydride anion is transferred from the *pro*‐S face of NADPH to the C17 position of Pchlide 60 and a conserved Tyr residue is proposed to donate a proton to the C18 position 61. Our previous laser photoexcitation studies have revealed that these two enzymatic H‐transfer reactions occur in a sequential mechanism on the microsecond timescale 12. By combining studies of the temperature and isotopic dependence, it was shown that both H‐transfer reactions proceed by quantum mechanical tunnelling and are coupled to promoting motions or vibrations in the enzyme–substrate complex 12. Moreover, a breakpoint at −27 °C in the temperature dependence of the hydride transfer rate suggests that motions/vibrations that are important for promoting light‐activated hydride tunnelling are quenched below −27 °C. We observed no such breakpoint for the proton tunnelling reaction, indicating a reliance on different promoting modes for this reaction in the enzyme–substrate complex 12.

**Figure 3:**
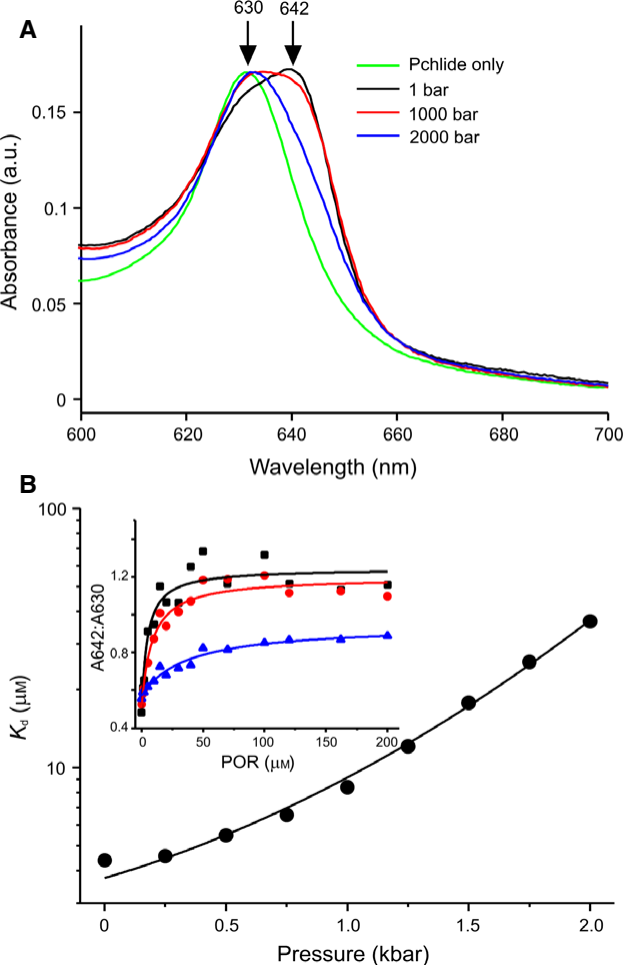
Pressure dependence of the ternary enzyme–substrate formation for POR. (A) Absorption spectra of the ternary complex formed from 7 μm Pchlide, 200 μm NADPH and 100 μm POR measured at 1 bar, 1 kbar and 2 kbar. Spectra were taken at room temperature and are compared with that of Pchlide measured at 1 bar. The arrows show the peak maxima for Pchlide and the red‐shift due to formation of the ternary complex. (B) The pressure dependence of the *K*_d_ for Pchlide. The data were fitted to Eqn (5) in the Materials and methods. (Inset) The level of ternary complex formation as a function of POR concentration at 1 bar (black), 1 kbar (red) and 2 kbar (blue). The data were fitted to a hyperbolic function to determine apparent *K*_d_ values.

**Figure 4:**
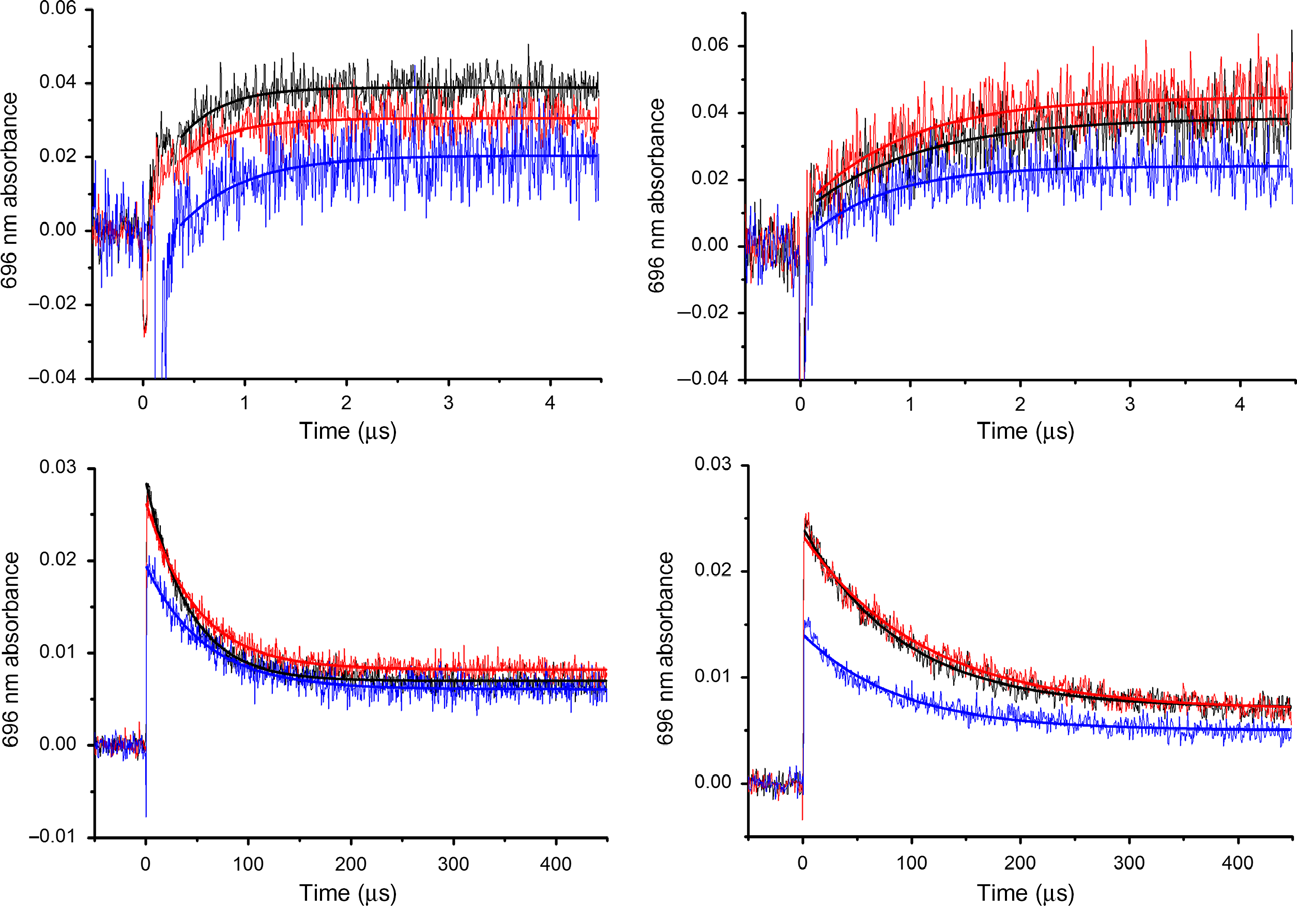
Representative POR photolysis transients measuring hydride transfer (upper, left), deuteride transfer (upper, right), proton transfer (lower, left) and deuteron transfer (lower, right) at 1 bar (black), 1 kbar (red) and 2 kbar (blue). Transients are fitted to a single exponential function (solid line), with averaged rate constants given in Table 1. Conditions: 50–260 μm POR (Table 1), 10 μm Pchlide, 500 μm NADPH, 0.1% 2‐mercaptoethanol, 0.1% Triton, 50 mm Tris, 150 mm NaCl, pH 7.5, 298 K. Deuteride transfer was measured using (*S*) ‐ [4‐^2^H]‐NADPH and deuteron transfer was measured in D_2_O buffer, pD 7.5.

**Figure 5:**
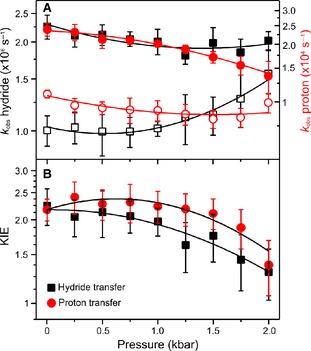
(A) Pressure dependencies of the observed rate constants for the sequential hydride and proton transfers catalysed by POR at 25 °C. The rate of hydride transfer from NADPH (black) and proton transfer in H_2_O (red) are indicated by filled symbols and the rate of deuteride transfer from *pro*‐*S* NADP ^2^H (black) and deuteron transfer in D_2_O are indicated by open symbols. (B) The pressure dependence of the resulting KIEs. The data have been fitted to Eqn (2), with fitting parameters given in Table 2 and the KIEs on these parameters given in Table 3. The rate constants are listed in Table 1.

We have now investigated the pressure dependence of both H‐transfer steps in POR to examine whether there are any inherent differences in the pressure dependencies of the proton and hydride transfer reactions. Because catalysis is triggered by photoexcitation of the dark‐assembled ternary complex of POR with NADPH and Pchlide, the pressure dependence of the POR–NADPH–Pchlide ternary complex formation was examined first (Fig. 3). It was found that the *K*_d_ for Pchlide increased significantly with increasing pressure (Fig. 3), likely because of the increased solvation of the active site at higher pressures. Stable ternary complexes were formed at all pressures studied (1 bar to 2 kbar) by using an excess of NADPH/D and POR over Pchlide and by varying the concentration of POR as a function of pressure (Table 1). The rate of hydride transfer was measured at a range of pressures between 1 bar and 2 kbar by following the increase in absorbance at 696 nm over 5 μs in the presence of NADPH and *pro*‐S NADP^2^H upon photoexcitation with a laser pulse at 450 nm (selective transients are shown in Fig. 4). There is a slight decrease in the hydride transfer rate at higher pressures in the presence of NADPH, whereas the rate for deuteride transfer increases at higher pressure (Fig. 5 and Table 1). The rate of the subsequent proton transfer reaction was measured at a range of pressures between 1 bar and 2 kbar by following the decrease in absorbance at 696 nm over 500 μs in both protiated and deuterated buffers. There is a more marked decrease in the proton transfer rate at higher pressures compared with hydride transfer, whereas there is a minimal pressure effect on the rate of deuteron transfer (Fig. 5 and Table 1). The data for both the hydride and proton transfer reactions were fitted to Eqn (2) to yield pressure‐dependent changes in the activation volume and activation isothermal compressibility (Table 2). The pressure dependence of the KIEs on the hydride and proton transfers catalysed by POR (Fig. 5 and Table 3) reveal that the isotope effect for both H‐transfer reactions decreases significantly at higher pressures. Hence, although there are no clear trends in the pressure dependencies of *k*_obs_, the pressure dependence of both KIEs are essentially superimposable, suggesting that there are unlikely to be large differences between the generic pressure response of hydride and proton transfers. If the pressure dependencies of the sequential KIEs on the POR‐catalysed reaction are dominated by distance compression rather than changes in vibrational coupling, the KIE data could be interpreted such that the reaction coordinate for both the hydride and proton transfers lay along a similar direction. Further analysis will likely require atomistic structural information (X‐ray crystal or NMR structure).

**Table 1:** Tabulated rate constants for the POR‐catalysed hydride and proton transfer data shown in Fig. 4

Pressure (bar)	[POR]^a^(μm)	*k*_obs_ hydride (s^−1^)		*k*_obs_ proton (s^−1^)	
		H	D	H	D
1	50	2.26 ± 0.21 × 10^6^	1.01 ± 0.12 × 10^6^	2.39 ± 0.18 × 10^4^	1.10 ± 0.05 × 10^4^
250	52	2.11 ± 0.18 × 10^6^	1.03 ± 0.13 × 10^6^	2.31 ± 0.23 × 10^4^	0.96 ± 0.08 × 10^4^
500	60	2.12 ± 0.17 × 10^6^	0.99 ± 0.17 × 10^6^	2.11 ± 0.21 × 10^4^	0.93 ± 0.07 × 10^4^
750	70	2.05 ± 0.12 × 10^6^	0.99 ± 0.12 × 10^6^	2.10 ± 0.10 × 10^4^	0.91 ± 0.14 × 10^4^
1000	85	2.01 ± 0.20 × 10^6^	1.02 ± 0.06 × 10^6^	2.02 ± 0.08 × 10^4^	0.90 ± 0.11 × 10^4^
1250	110	1.80 ± 0.12 × 10^6^	1.11 ± 0.21 × 10^6^	1.89 ± 0.09 × 10^4^	0.86 ± 0.11 × 10^4^
1500	156	1.99 ± 0.25 × 10^6^	1.14 ± 0.17 × 10^6^	1.71 ± 0.12 × 10^4^	0.81 ± 0.09 × 10^4^
1750	200	1.85 ± 0.20 × 10^6^	1.29 ± 0.26 × 10^6^	1.55 ± 0.17 × 10^4^	0.83 ± 0.10 × 10^4^
2000	260	2.02 ± 0.15 × 10^6^	1.56 ± 0.31 × 10^6^	1.36 ± 0.26 × 10^4^	0.99 ± 0.12 × 10^4^

a The enzyme concentration used to maintain a saturated ternary complex (see Fig. 3).

**Table 2:** Pressure dependencies of the rate of hydride and proton transfer catalysed by POR

	Hydride		Proton	
	H	D	H	D
*k*_0_ (s^−1^)	(2.30 ± 0.09) × 10^6^	(1.03 ± 0.02) × 10^6^	(2.35 ± 0.04) × 10^4^	(1.08 ± .03) × 10^4^
Δ*V* ^‡^ (cm^3^·mol^−1^)	6.6 ± 2.2	5.0 ± 1.2	1.7 ± 1.0	7.5 ± 2.3
Δβ^‡^ (cm^3^·mol^−1^·kbar^−1^)	4.7 ± 2.0	9.5 ± 1.3	−4.7 ± 1.1	4.9 ± 2.5

**Table 3:** Pressure and temperature dependencies of selected 1° KIEs on biological H‐transfers. Data were taken from parameters obtained by fitting Eqns (1) and (2) to experiments performed at 293–298 K. Systems are: MR RHR with NADH 11, 23; PETNR RHR with NADH and NADPH 30; AADH with phenylethylamine 46; POR 12 and this work; ascorbate oxidation by ferricyanide ± tetraethylammonium chloride 55

System	KIE_0_	ΔΔ*H* ^‡^ (kJ·mol^−1^)	ΔΔ*S* ^‡^ (J·mol^−1^·K^−1^)	ΔΔ*V* ^‡^ (cm^3^·mol^−1^)	ΔΔβ^‡^ (cm^3^·mol^−1^·kbar^−1^)
MR	6.8 ± 0.1	7.2 ± 1.5	20 ± 13	−8.8 ± 1.3	4.8 ± 1.1
PETNR, NADPH	7.0 ± 0.1	6.5 ± 2.8	7 ± 6	0.6 ± 6.1	2.6 ± 6.5
PETNR, NADH	8.1 ± 0.1	−1.1 ± 2.1	−20 ± 6	5.4 ± 3.0	−1.9 ± 3.2
AADH	12.8 ± 0.8	6.8 ± 3.9	1.6 ± 13.5	1.9 ± 2.7	0.9 ± 2.3
POR, hydride	2.2 ± 0.1	8.2 ± 0.6	21.2 ± 3.3	1.6 ± 3.3	−4.8 ± 3.3
POR, proton	2.2 ± 0.1	9.8 ± 0.8	26.5 ± 1.2	−5.8 ± 3.3	−9.6 ± 3.6
Asc, FeCyn	5.1 ± 0.1	1.1 ± 0.9	−10 ± 3	7.5 ± 0.4	0^a^
Asc, FeCyn, TEA	9.9 ± 0.1	6.1 ± 0.9	2 ± 3	1.8 ± 1.4	0^a^

a Data fitted with Δβ fixed to 0.

## Correlating the pressure and temperature of KIEs

**Figure 6:**
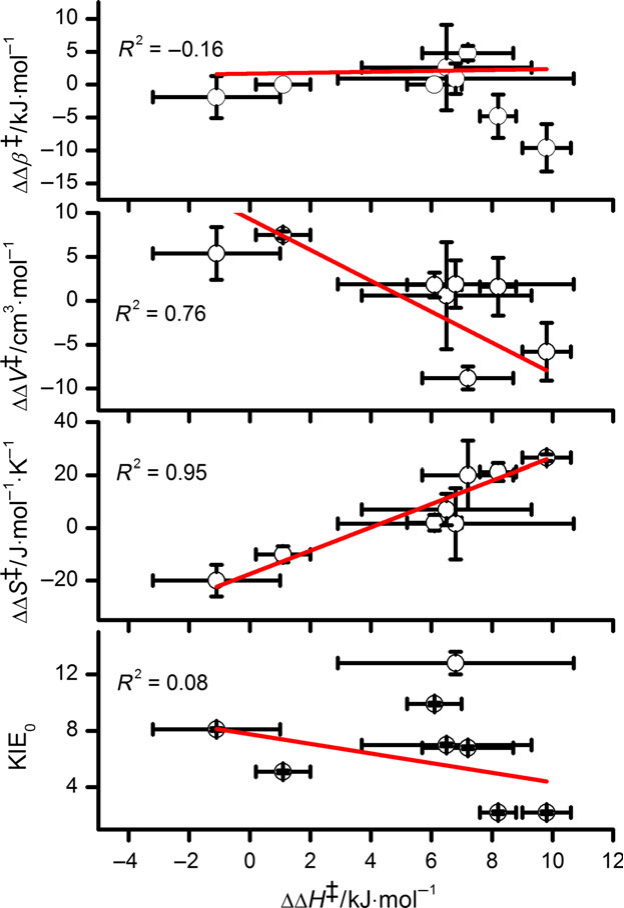
Linear correlations of ΔΔ*H* ^‡^ with ΔΔβ^‡^, ΔΔ*V* ^‡^, ΔΔ*S* ^‡^ and KIE_0_ for the data in Table 3. Adjusted *R* ^2^ values were determined for the error‐weighted linear fits as shown.

As described above, the *p–T* dependence of a KIE can be largely described by the five parameters: ΔΔ*H* ^‡^, ΔΔ*S* ^‡^, KIE_0_, ΔΔ*V* ^‡^ and ΔΔβ^‡^. These parameters are listed in Table 3 for the MR, PETNR, AADH, ascorbate and POR experiments described above. We are not aware of other enzyme/biological systems in which both the temperature and pressure dependence of the KIE have been measured, but Table 3 should not be taken to be exhaustive. Because the focus of this work is to determine whether pressure offers an alternative probe of the temperature dependence of KIEs, we examined the correlation of ΔΔ*S* ^‡^, KIE_0_, ΔΔ*V* ^‡^ and ΔΔβ^‡^ with ΔΔ*H* ^‡^ (Fig. 6). ΔΔ*S* ^‡^ shows a strong linear correlation with ΔΔ*H* ^‡^, a trend we have previously demonstrated in the context of Arrhenius parameters; ΔΔ*S* ^‡^ ~ *R*ln(–*A* ^H^/*A* ^D^), and we showed that ln(*A* ^H^/*A* ^D^) is a linear function of Δ*E*_a_ for the KIE on the RHRs of a range of isotopically substituted PETNR enzymes 40.

There is some correlation between ΔΔ*V* ^‡^ and ΔΔ*H* ^‡^, with more temperature‐dependent KIEs exhibiting more negative ΔΔ*V* ^‡^ values (Fig. 6). However, there is no correlation between ΔΔβ^‡^ or KIE_0_ and ΔΔ*H* ^‡^. In the context of Eqn (4), ΔΔ*V* ^‡^ and ΔΔβ^‡^ report on the change with pressure in the vibrational coupling (κ) and distance sampling (*r*), respectively, of the transferred H isotope 23. Although this is a simplification, temperature‐dependent KIEs have been used as evidence of the vibrational coupling of protein dynamics to the reaction coordinate 9, 10, 11, 12, so the correlation of ΔΔ*V* ^‡^ with ΔΔ*H* ^‡^ is not unexpected.

### Trends/outlooks/unifying concepts

To date, fast dynamics in enzymes have largely been inferred from the anomalous temperature dependencies of KIEs on H‐transfer reactions 9, 10, 11, 12. However, the atomistic origin of the, sometimes large, temperature dependencies of KIEs remains unresolved, and alternative probes of fast enzyme dynamics are desirable. Hydrostatic pressure offers a fairly convenient and unique method to perturb existing equilibria in chemical systems including enzymes, and we have demonstrated that a range of KIEs on enzyme‐catalysed and biochemical reactions are pressure dependent (Table 3). However, there is no clear trend between the pressure and temperature dependencies of the KIEs we have examined (Fig. 6), suggesting that the atomistic origin of these two perturbations are not equivalent. Coupled with this atomistic understanding of pressure responses, studies of the *p*‐*T* dependence of reaction rates and KIEs provide rich new datasets from which to infer the importance (or otherwise) of dynamics coupled to the reaction coordinate. These datasets should be considered on a case‐by‐case basis, where they can support/enrich the more generalized conclusions that have emerged from temperature‐dependence studies of KIEs in relation to enzymatic H‐transfer.

## Materials and methods

Both recombinant POR from *Thermosynechococcus elongatus* and Pchlide were prepared as described previously 62. (*S*) ‐ [4‐^2^H] ‐NADPH was prepared and characterized as previously 49. All high‐pressure experiments were performed using a high‐pressure cell system (ISS Inc., Champaign, IL, USA) with the sample contained in cylindrical cuvette bottles with a 1 cm path length. Absorbance spectra were measured using a Cary 50 spectrometer (Agilent Technologies, Santa Clara, California, USA) with the pressure cell mounted using a custom‐made platform. Upon binding to the enzyme, the peak maxima of Pchlide becomes red‐shifted from 630 to 642 nm (Fig. 1A). The *K*_d_ for Pchlide was determined by measuring the ratio of the absorbance peaks at 642 and 630 nm at increasing concentrations of POR and fitted to a hyperbolic function. Measurements were repeated at a range of pressures from 1 to 2000 bar in 250 bar increments. Samples contained 10 μm Pchlide, 200 μm NADPH and 0–200 μm POR in 50 mm Tris pH 7.5, 150 mm NaCl, 0.1% 2‐Mercaptoethanol, 0.1% Triton X‐100. The pressure dependence of the *K*_d_ for Pchlide (Fig. 3) was determined by fitting to Eqn (5):

(5) $$K_{d}\left(p\right)=K_{d,0}\exp\left(-\Delta Vp/RT\right)\exp\left(\Delta \beta p^{2}/2RT\right)$$

where *K*_d,0_ is the Pchlide *K*_d_ at 0 bar (3.7 ± 0.4 μm), Δ*V* is the activation volume (−15.9 ± 3.6 cm^3^·mol^−1^) and Δβ is the compressibility (12.5 ±2.5 cm^3^·mol^−1^·kbar^−1^).

Laser photolysis experiments were performed essentially as described in Heyes *et al*. 12. Briefly, rate constants for the POR‐catalysed hydride and proton transfer were measured using laser photoexcitation of the dark‐assembled ternary complex (POR–NADPH–Pchlide) at 25 °C. Samples were made up to a total volume of 1300 μL, comprising 50–260 μm POR (increasing concentration at higher pressures), 320 μm NADPH and 15 μm Pchlide in 50 mm Tris pH 7.5, 150 mm NaCl, 0.1% (v/v) 2‐mercaptoethanol, 0.1% Triton X‐100. Samples were excited at 450 nm using an optical parametric oscillator of a Q‐switched Nd–YAG laser (Brilliant B, Quantel) that produces between 6 and 8 ns laser pulses (30 mJ). The detection system is an LKS‐60 flash photolysis instrument (Applied Photophysics Ltd., Leatherhead, UK) and is at a right angle to the incident laser beam. Transients were collected at 696 nm and were fitted to a single exponential to obtain rate constants for the hydride and proton transfer reactions (Fig. 4). Typically, each data point is an average of two or three separate samples, with two or three shots measured per sample.

## Author contributions

NSS and DJH Planned experiments; RH and DJH Performed experiments; DJH, SH and NSS Analyzed data; All authors wrote the paper.
